# iTRAQ-based proteome profile analysis of superior and inferior Spikelets at early grain filling stage in japonica Rice

**DOI:** 10.1186/s12870-017-1050-2

**Published:** 2017-06-07

**Authors:** Cuicui You, Lin Chen, Haibing He, Liquan Wu, Shaohua Wang, Yanfeng Ding, Chuanxi Ma

**Affiliations:** 1 College of Agronomy, Anhui Agricultural University, Hefei, 230036 People’s Republic of China; 2 College of Agronomy, Nanjing Agricultural University/Key Laboratory of Crop Physiology Ecology and Production Management, Ministry of Agriculture, Nanjing, 210095 People’s Republic of China; 3 Jiangsu Collaborative Innovation Center for Modern Crop Production, Nanjing, 210095 People’s Republic of China

**Keywords:** Rice, Removal of superior spikelets, Inferior spikelets, Grain filling, iTRAQ, Proteome

## Abstract

**Background:**

Large-panicle rice varieties often fail to achieve their yield potential due to poor grain filling of late-flowering inferior spikelets (IS). The physiological and molecular mechanisms of poor IS grain filling, and whether an increase in assimilate supply could regulate protein abundance and consequently improve IS grain filling for japonica rice with large panicles is still partially understood.

**Results:**

A field experiment was performed with two spikelet removal treatments at anthesis in the large-panicle japonica rice line W1844, including removal of the top 1/3 of spikelets (T1) and removal of the top 2/3 of spikelets (T2), with no spikelet removal as a control (T0). The size, weight, setting rate, and grain filling rate of IS were significantly increased after spikelet removing. The biological functions of the differentially expressed proteins (DEPs) between superior and inferior spikelets as well as the response of IS to the removal of superior spikelets (SS) were investigated by using iTRAQ at 10 days post anthesis. A total of 159, 87, and 28 DEPs were identified from group A (T0-SS/T0-IS), group B (T0-SS/T2-IS), and group C (T2-IS/T0-IS), respectively. Among these, 104, 63, and 22 proteins were up-regulated, and 55, 24, and 6 proteins were down-regulated, respectively. Approximately half of these DEPs were involved in carbohydrate metabolism (sucrose-to-starch metabolism and energy metabolism) and protein metabolism (protein synthesis, folding, degradation, and storage).

**Conclusions:**

Reduced endosperm cell division and decreased activities of key enzymes associated with sucrose-starch metabolism and nitrogen metabolism are mainly attributed to the poor sink strength of IS. In addition, due to weakened photosynthesis and respiration, IS are unable to obtain a timely supply of materials and energy after fertilization, which might be resulted in the stagnation of IS development. Finally, an increased abundance of 14–3-3 protein in IS could be involved in the inhibition of starch synthesis. The removal of SS contributed to transfer of assimilates to IS and enhanced enzymatic activities of carbon metabolism (sucrose synthase, starch branching enzyme, soluble starch synthase, and pullulanase) and nitrogen metabolism (aspartate amino transferase and alanine amino transferase), promoting starch and protein synthesis in IS. In addition, improvements in energy metabolism (greater abundance of pyrophosphate-fructose 6-phosphate 1-phosphotransferase) might be played a vital role in inducing the initiation of grain filling. These results collectively demonstrate that carbohydrate supply is the main cause of poor IS grain filling.

**Electronic supplementary material:**

The online version of this article (doi:10.1186/s12870-017-1050-2) contains supplementary material, which is available to authorized users.

## Background

Rice is a major staple food crop worldwide, and its consumption is increasing as the world’s population grows. Improving the output per unit area is therefore necessary for producing more rice on a limited land area [1]. Many efforts have been made to reach this target by breeders, who have attempted to expand the sink capacity by increasing the number of spikelets per panicle, creating extra-heavy panicle types or large-panicle rice varieties [2]. These cultivars with larger sink capacities, however, generally do not produce the expected yield due to the low seed setting rate and grain weight of inferior spikelets (IS) [3, 4]. Within the rice panicle, spikelets are grouped into superior spikelets (SS) and IS according to their location on the branch and the time of flowering [5]. Generally, SS are located on upper primary branches, and they flower earlier, fill more quickly, and produce larger and heavier grains. The IS are located on the lower secondary branches, and they flower later, fill more slowly, and produce smaller grains [6]. Therefore, improving IS grain filling is important for achieving a high yield potential of large-panicle rice varieties.

There are many explanations that may account for poor IS grain filling, including reduced activities of the enzymes involved in sucrose-to-starch conversion [7, 8], hormone imbalances [4], assimilate transportation obstacles [3, 9], and the differential expression of genes associated with cell growth and signal transduction [10]. However, whether the assimilate supply is a crucial factor for poor IS grain filling remains controversy [3, 6, 11]. In order to clarify this question, previous studies have normally used leaf- and flower-thinning methods to regulate the source-sink balance [12, 13]. For example, Xu et al. [14] found that IS grain weight and grain setting rate were significantly increased following removal of SS. However, Kato [15] reported that IS filling was not significantly improved by SS removal. Our previous study showed that SS removal could force assimilate transport to IS, promoting IS grain filling, and the possible physiological mechanisms underlying this process have been discussed [16]. However, rice grain filling is a highly complex biological process, and previous studies have primarily focused on the relationship between spikelet removal and grain weight or the underlying physiological mechanisms. Thus, the effect of SS removal on protein abundances in IS and how these interact with IS grain filling remains unclear.

In recent years, proteomics has become an essential technique for revealing the mechanisms of poor IS grain filling. Proteomics contributes to a greater understanding of complex biological systems as it allows for the simultaneous analysis of changes in multiple proteins [17]. There have been numerous studies that have attempted to resolving the problem of poor IS grain filling by reporting differences in protein abundance between SS and IS. Zhang et al. [18] employed two-dimensional gel electrophoresis (2-DE)-based comparative proteomic and phosphoproteomic analyses to explore differentially expressed proteins in IS following spraying with abscisic acid (ABA); a total of 111 differentially expressed proteins (DEPs) were found to be associated with defense response, carbohydrate, protein, amino acid, energy, secondary metabolism, cell development, and photosynthesis, demonstrating that IS grain filling was improved by ABA through proteins and phosphoproteins that participate in carbon, nitrogen, and energy metabolisms. Furthermore, Zhang et al. [19] reported that the 14–3-3 protein plays an important role in the signaling networks of IS development, especially in developmental stagnancy. Chen et al. [20] also compared differential protein expression between SS and IS using the 2-DE method and found that the dramatic down-regulation of functional proteins related to photosynthesis, carbohydrate and energy metabolism, amino acids metabolism, and defense responses was the main cause of poor IS grain filling. In addition, they found that post-anthesis alternate wetting and moderate soil drying could improve grain filling by regulating protein expression, especially in IS. Although 2-DE could separate thousands of different proteins and provide visual information of the proteome including distinct protein isoforms resulting from changes in Mr. (relative molecular mass) and pI (isoelectric point), it is not suitable for detection of low-abundance proteins and more accurate quantification. Isobaric tags for relative and absolute quantitation (iTRAQ) is a mass spectrometry-based quantitative approach that has become prevalent in developmental grain proteomics, as it simultaneously identifies and quantifies proteins from multiple samples with high coverage [21]. It has been reported that lower sink strength and smaller sink sizes result in reduced decomposition, conversion of photoassimilate, and slower cell division in hybrid rice [22]. However, previous studies were only made on hybrid rice or an indica varieties, and little proteomic information has documented using iTRAQ regarding SS and IS and the response of IS to SS removal in homozygous japonica rice.

This study investigated whether an increase in assimilate supply could regulate protein abundance and consequently improve IS grain filling for japonica rice with large panicles. Transfer of assimilates toward IS was forced by removal of SS, and we examined subsequent changes in grain weight, seed setting rate, and grain filling rate of IS during the grain filling period. Additionally, iTRAQ technology was used to identify DEPs between SS and IS under different treatments and their biological functions, and then we analyzed the relationship between these proteins and grain development to reveal the underlying causes of differences in grain filling between SS and IS as well as the response of IS to SS removal at proteomic level.

## Methods

### Plant materials

The experiment was conducted in 2015 at the Danyang Experimental Base of the Nanjing Agricultural University, Jiangsu Province, China (31°54′31″N, 119°28′21″E) during the rice growing season. In order to analyze the mechanisms of poor IS grain filling at the molecular level, the experiment was conducted using the homozygous large-panicle japonica rice line W1844, which is an inbred line and not a hydrid or transgenetic line. Moreover, W1844 is the intermediate material of breeding, but its genetic characteristics have stabilized. The seeds of W1844 were provided by the professor jian-min wan of the State Key Laboratory of Rice Genetics and Germplasm Innovation, Nanjing Agricultural University, Jiangsu, China. W1844 has 265 grains per panicle and thus is typical of large-panicle rice varieties. Its plant height, panicle length, thousand-grain weight, and seed setting rate are 99.8 cm, 18.1 cm, 23.6 g, and 92.1% respectively. Seeds were sown on May 28th, 2015, and seedlings were transplanted to the field on June 18th at a hill spacing of 13.3 cm × 30 cm. The trials were designed in randomized plots with three replicates, and each plot was 5 m × 10 m. The soil type was clay loam, and 280 kg·ha^−1^ nitrogen was applied during the growing season. Nitrogen fertilizer was converted into urea according to the nitrogen content and was applied according to the ratio of base fertilizer to panicle fertilizer (5:5). The base fertilizer was applied before seedling transplantation, and the panicle fertilizer was applied when the leaf-age remainder was 3.5. Cultivation and management measures were applied according to the technical requirements of the local high-yield field.

### Experimental design

A total of 800 single stems (panicles) with similar growth patterns that flowered on the same day were labeled during heading-blooming stage. Once most labeled panicles had withdrawn from flag leaf sheath completely, two spikelet thinning treatments were performed: T0 was control treatment with no spikelet thinning, T1 plants had the upper 1/3 of spikelets removed, and T2 plants had the upper 2/3 of spikelets removed. Spikelet thinning involved removal of the primary branch. The primary branches of each panicle were equally divided into three parts: upper, middle, and lower. If the number of primary branches could not be divided equally, a number of spikelets equal to the integer of the average branch number was included in each of the upper and lower parts, and the remaining branches were included in the middle part. SS were considered to be the grains on the three primary branches on the upper part of the panicle, while medium spikelets (MS) were defined as the grains on the three primary branches in the middle part of the panicle, and IS were the grains on the three primary branches in the lower part.

### Sampling and measurement

#### Determination of the grain filling rate

From anthesis to maturity, 50 tagged panicles from each plot were collected every 5 days. SS, MS, and IS samples were collected from the T0 group; MS and IS were collected from the T1 group, and only IS were collected from the T2 group. Two-fifths of the sampled grains were frozen in liquid nitrogen and stored at −80 °C for protein extraction. The remaining grains were deactivated at 105 °C for 0.5 h and dried at 80 °C until they reached a constant weight. They were then weighed to determine grain dry weights. Richards’s growth eq. [23] was used for grain filling process fitting and grain filling rate calculation:

1 $$ W=\frac{A}{{\left(1+{Be}^{- kt}\right)}^{1/ N}} $$

The grain filling rate (R) was calculated as the derivative of the Eq. (1)

2 $$ R=\frac{AkBe^{- kt}}{N{\left(1+ Be{-}^{\mathrm{kt}}\right)}^{\left( N+1\right)/ N}} $$

where *W* is the grain weight (mg); *A* is the final grain weight (mg); *t* is the time after anthesis (days); and *B*, *k*, and *N* are coefficients established from the regression of the equation.

#### Protein extraction

Protein extraction was performed according to Isaacson et al. [24] with some modifications. About 0.1 g dehulled grains were homogenized with a pestle in a pre-cooled mortar containing ice-cold 10% (*w*/*v*) trichloroacetic acid in acetone. They were incubated at −20 °C for 1 h, followed by centrifugation at 15000 g for 15 min at 4 °C in a refrigerated high-speed centrifuge, after which the precipitate was collected. After vacuum drying, adding an equal volume of phenol saturated with Tris-HCl (pH 7.5), and centrifugation at 5000 g for 30 min at 4 °C, we collected the upper phenolic phase. Five volumes of pre-cooled 0.1 M ammonium acetate in methanol were added to collected phenol phase, followed by centrifugation at 10000 g for 10 min at 4 °C, after which the precipitate was collected; this process was repeated three times. Protein concentration was determined by the BCA method [25].

#### Protein digestion and iTRAQ labeling

Protein digestion was performed according to the method of FASP [26]. Five volumes of cold acetone were added to 100 μg protein from each sample and centrifuged at 12000 rpm for 10 min at 4 °C, collected the precipitate and dried by speed vacuum concentrator. 50 μL dissolution buffer was added for dissolve protein precipitation, and added 4 μL reducing reagent, incubated at 60 °C for 1 h, then added 2 μL cysteine-blocking reagent at room temperature for 10 min. Clean the protein solution by using 10 KDa ultrafiltration tube to centrifuge at 12000 rpm for 20 min, and discarded the solution at the bottom of the collection tube; 100 μL dissolution buffer was added to the ultrafiltration tube, then centrifuged at 12000 rpm for 15 min, discarded the solution at the bottom of the collection tube and repeat this step three times. Replace a new collection tube, 50 μL sequencing-grade trypsin (50 ng/μL) was placed into the ultrafiltration tube, incubated at 37 °C for 12 h, and centrifuged at 12000 rpm for 20 min, then collected the peptides. Transfered the filter units to new collection tube and added 50 μL dissolution buffer to centrifuge the tube again, and combined the two filter solution, which contained peptides. The peptides were dried in a centrifugal speed vacuum concentrator.

Two biological replicates were performed for each sample for iTRAQ analysis. The peptides of each sample were labeled using iTRAQ 8-plex kits according to the manufacturer’s manual (AB SCIEX Inc., USA). Labelling was performed by adding one reagent vial, containing an isobaric tag, to 110 μg of dried peptides for each sample. The labelling reaction proceeded for 3 h at room temperature after which all the samples were pooled before application of separation techniques and mass spectrometry analysis. The labelling scheme was as follows: Tags 113 and 117, T0-SS; Tags 114 and 118, T0-IS; Tags 115 and 119, T2-IS.

#### Two dimensional liquid chromatography tandem mass spectrometry (2D–LC-MSMS) analysis

After labeling, all samples were pooled and purified using a strong cation exchange chromatography (SCX) column by Agilent 1200 HPLC (Agilent). The HPLC column was purchased from Agilent, and its parameters were as follows: the Analytical Guard Column 4.6 × 12.5 mm 5-Micron; Narrow-Bore 2.1 × 150 mm 5 μm with 215 nm and 280 nm UV detection. Separation was performed at 0.3 mL/min using a nonlinear binary gradient. Collected the first peptides from 0 to 5 mins, then collected each peptides with 4.5 mins interval for the 6–45 min, and for the last peptides from 46 to 50 mins, with a total of 10 peptides. Dried every peptides in a vacuum freezed dryer for LC-MSMS Analysis.

The dried peptides were re-suspended with Nano-RPLC buffer A (0.1% formic acid, 2% acetonitrile). The online Nano-RPLC was employed on the Eksigent nanoLC-Ultra™ 2D System (AB SCIEX). The samples were loaded on C18 NanoLC trap column (100 μm × 3 cm, C18, 3 μm, 150 Å) and washed by Nano-RPLC Buffer A at 2 μL/min for 10 mins. An elution gradient of 5–35% acetonitrile (0.1% formic acid) in 70 mins gradient was used on an analytical ChromXP C18 column (75 μm × 15 cm, C18, 3 μm 120 Å) with spray tip. The LC fractions were analyzed using a Triple TOF 5600 mass spectrometer.

Mass spectrometer data acquisition was performed with a Triple TOF 5600 System (AB SCIEX, USA) fitted with a Nanospray III source (AB SCIEX, USA) and a pulled quartz tip as the emitter (New Objectives, USA). Data were acquired using an ion spray voltage of 2.5 kV, curtain gas of 30 PSI, nebulizer gas of 5 PSI, and an interface heater temperature of 150 °C. For information dependent acquisition (IDA), survey scans were acquired in 250 ms and as many as 35 product ion scans were collected if they exceeded a threshold of 150 counts per second (counts/s) with a 2^+^ to 5^+^ charge-state. The total cycle time was fixed to 2.5 s. A rolling collision energy setting was applied to all precursor ions for collision-induced dissociation (CID). Dynamic exclusion was set for 1/2 of peak width (18 s), and the precursor was then refreshed off the exclusion list.

#### Bioinformatics analysis

The proteins were identified in two biological replicates using the iTRAQ technique. Data were processed with Protein Pilot Software v. 5.0 (AB SCIEX, USA) against *Oryza sativa* database of UniProt using the Paragon algorithm [27]. The experimental data from tandem mass spectrometry were matched against theoretical data for protein identification. The iTRAQ 8-plex was chosen for protein quantification with unique peptides during the search. According to the abundances of proteins and the results of comparison among groups, the screening criteria for authentic proteins was an FDR ≤ 1% and a unique peptide ≥1. The screening criteria for DEPs was a fold change >1.5 or <0.67 and a *p*-value <0.05. The bioinformatics data analysis tool, OmicsBean, was used to analyze the obtained proteomics data (http://www.omicsbean.cn/), in which distributions in biological functions, cell component and molecular functions were assigned to each protein based on Gene Ontology (GO) categories. The Kyoto Encyclopedia of Genes and Genomes (KEGG) pathway analysis was performed in order to enrich high-level functions in the defined biological systems.

#### Assessment of panicle characteristics

Approximately 90 tagged panicles from each treatment were harvested at maturity. The SS, MS, and IS were collected from T0 group, MS and IS were collected from T1 group, and IS were collected from T2 group. The samples were naturally dried, and the grain weight and seed setting rate were measured. The seed setting rate was determined using the method of Kobata et al. [28].

#### Statistical analysis

For all statistical analyses, at least three biological replicates were used for each treatment and control. Statistical analyses of the data were accomplished by the standard analysis of variance (ANOVA) and mean values were tested by least significant difference (LSD) at the 5% level using SPSS16.0.

## Results

### Grain weight and grain setting rate

Grain weights and seed setting rates were significantly different among the SS, MS, and IS of W1844 under T0 treatment, while SS has the highest and IS has the lowest values (Table 1). Compared with T0 group, the grain weights and seed setting rates of MS and IS in T1 group, as well as those of the IS in T2 group were all increased, with the greatest improvement in seed setting under T2 treatment. Therefore, in the subsequent analyses, we focused on the effect of T2 treatment on IS grain filling. As shown in Table 1, the grain weight and seed setting rate of IS under T2 were the same as those of SS under T0, demonstrating that SS removal significantly improved IS grain weight and seed setting rate.

**Table 1:** Grain weight and seed setting rate under different treatments

Treatments	T0			T1		T2
	Superior	Medium	Inferior	Medium	Inferior	Inferior
Grain weight (mg/grain)	26.6 ± 0.13a	23.2 ± 0.53c	20.9 ± 0.39d	26.2 ± 0.23b	25.6 ± 0.45b	28.2 ± 0.12a
Seed setting rate (%)	97.1 ± 0.47a	93.5 ± 0.39b	85.7 ± 0.26c	96.8 ± 0.26a	92.5 ± 1.10b	96.6 ± 0.76a

T0: control treatment with no spikelet thinning; T1: the upper 1/3 of spikelets were removed; T2: the upper 2/3 of spikelets were removed. Values are means ± S.D. of three replications. The different lowercase letters labeled after the data from the same character indicate significant differences at the 0.05 level

### Grain filling of SS and IS

**Fig. 1:**
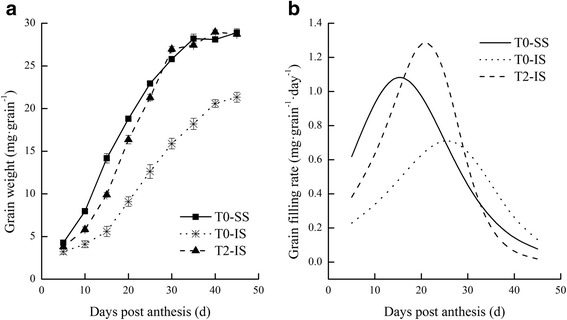
Grain weight and grain filling rate of SS and IS of rice during grain-filling period. T0 represent control treatment with no spikelet thinning and T2 represent treatment with the upper 2/3 of spikelets were removed. The *black square* represent superior spikelets under the T0 treatment, the asterisk represent inferior spikelets under the T0 treatment, and the *black triangle* represent inferior spikelets under the T2 treatment. *Vertical bars*, where values exceed size of symbol, represent ± SEM (*n* = 3)

The dynamic changes in grain weight and grain filling rate in W1844 during the grain filling period were shown in Fig. 1. The IS grain weight was consistently lower than that of SS throughout the filling process, while under T2 treatment, the IS grain weight began to increase and reached the SS level at 30 days post anthesis (DPA) (Fig. 1-a). We found that the initial and maximum grain filling rates of IS were consistently lower than those of SS, and peak grain filling also appeared later in IS than that in SS (Fig. 1-b). Compared with T0 treatment, T2 treatment significantly increased the initial and maximum grain filling rates of IS. Moreover, the peak value of IS grain filling rate under T2 was higher and occurred 5 days earlier than that of IS in T0 group. Changes in grain weight and grain filling rate indicated that removal of SS significantly improved IS grain filling.

### Grain morphology of SS and IS

**Fig. 2:**
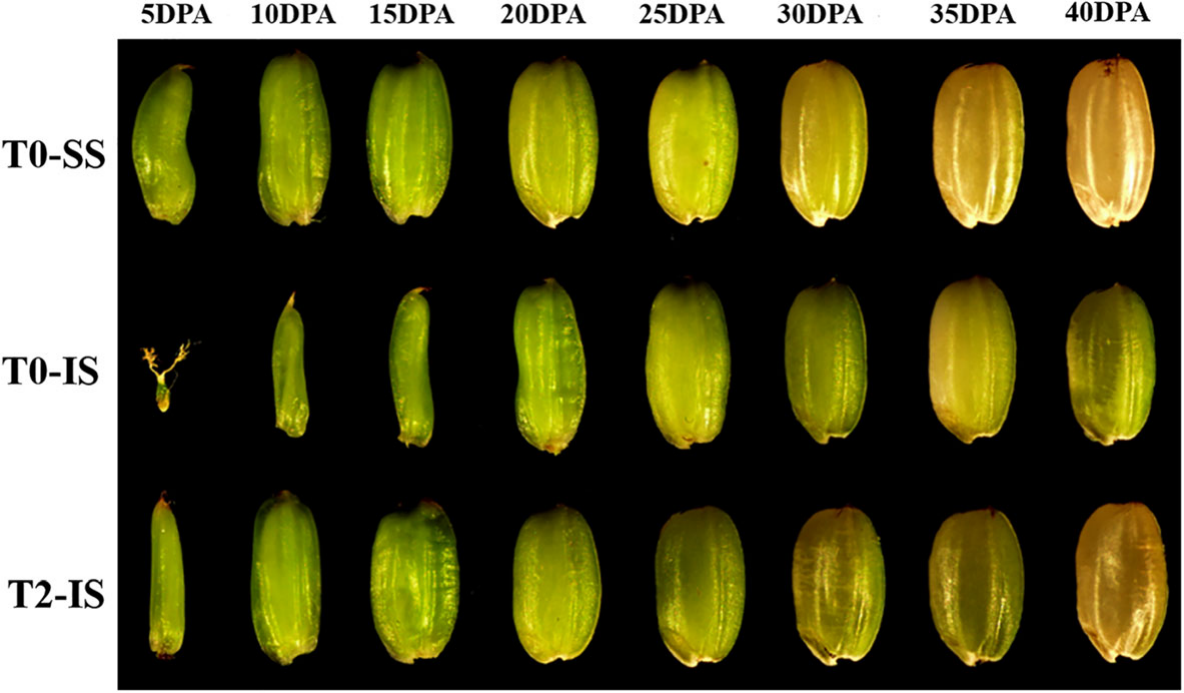
The morphology of SS and IS in rice during grain filling period under different treatments (observed under stereoscope × 6.3). T0 represent control treatment with no spikelet thinning and T2 represent treatment with the upper 2/3 of spikelets were removed

Changes in the kernel development dynamics of SS and IS under different treatments are shown in Fig. 2. We observed that the SS first elongated and then widened after flowering, and SS grain size showed a rapid increase. However, the IS developed slowly during the early stage of grain filling (days 5–15), and its grain morphology changed greatly at 20 DPA. Compared to IS under T0, grain size and grain weight of IS under T2 treatment increased significantly at 10 DPA (Fig. 1-a), indicating that important changes occurred within the kernel during this time and affected the development of the IS. Some studies have shown that the physiological activities of grain are significantly positively correlated with grain filling at the beginning of the filling stage [29, 30]. Therefore, the subsequent experiment studied protein expression in the grains under different treatments at 10 DPA.

### DEPs in SS and IS at 10 DPA under different treatments

In order to further study the reason behind the grain filling difference between SS and IS, as well as molecular mechanism of IS response to SS removal, we used comparative proteomics to analyze protein expression in SS and IS. A total of 4631 proteins were identified in two biological replicates using the iTRAQ technique and were subjected to comparative analysis. Protein abundances that changed by more than 1.5-fold or less than 0.67-fold were selected. Following this criterion, a total of 174 types of proteins were detected which showed that there were differentially abundant between SS and IS under different treatments at 10 DPA.

**Fig. 3:**
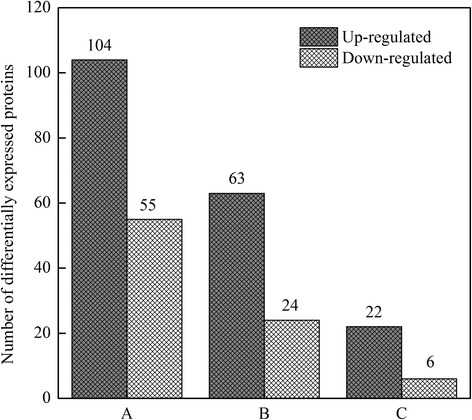
Patterns of change on differentially expressed proteins of **a** (T0-SS / T0-IS), **b** (T0-SS / T2-IS) and **c** (T2-IS / T0-IS). T0 represent control treatment with no spikelet thinning and T2 represent treatment with the upper 2/3 of spikelets were removed

Table 2 lists these DEPs between SS and IS under different treatments, providing the accession numbers and names of these proteins according to the Uniprot database as well as their fold changes in abundance. The numbers of DEPs and their changes in abundance are listed in Fig. 3. As Fig. 3 shows, in the T0-SS/T0-IS comparison, 159 DEPs were identified, of which 104 proteins (65.4%) were up-regulated and 55 proteins (34.6%) were down-regulated; in the T0-SS/T2-IS comparison, 87 DEPs were identified, of which 63 proteins (72.4%) were up-regulated and 24 proteins (27.6%) were down-regulated; and in the T2-IS/T0-IS comparison, 28 DEPs were identified, of which 22 proteins (78.6%) were up-regulated and 6 proteins (21.4%) were down-regulated.

**Table 2:** Identification of 159, 87 and 28 differentially expressed proteins (≥ 1.5 fold) between SS and IS at 10 DAP in group A (T0-SS/T0-IS), B (T0-SS/T2-IS) and C (T2-IS/T0-IS)

Protein No.	Accession	Uniprot date accession no.	Protein name UniprotKB database	Fold-change (≥1.5-fold)		
				T0-SS/T0-IS (A)	T0-SS/T2-IS (B)	T2-IS/T0-IS (C)
Cell growth/division						
36	Os02g0753800	Q6Z6A7	Annexin	3.01 ± 0.71	ns	ns
91	Os05g0438800	Q75HX0	Actin	2.89 ± 0.12	1.76 ± 0.08	ns
154	Os07g0249700	Q8H3C8	IAA-amino acid hydrolase ILR1-like 8	3.52 ± 0.47	2.06 ± 0.52	ns
Sugar metabolism						
14	Os03g0758100	Q9AUV8	Alpha-1,4 glucan phosphorylase	4.70 ± 0.48	3.18 ± 1.26	ns
19	Os06g0194900	P30298	Sucrose synthase 2	6.04 ± 1.05	ns	3.57 ± 1.79
26	Os03g0278000	Q8W3J0	Os03g0278000 protein	0.35 ± 0.04	ns	ns
33	Os03g0703000	Q75I93	Beta-glucosidase 7	0.40 ± 0.02	ns	ns
38	Os01g0944700	Q94CR1	Beta 1,3-glucanase	2.82 ± 0.20	2.45 ± 0.27	ns
50	Os10g0340600	Q7XFK2	Beta-galactosidase 14	0.40 ± 0.13	ns	ns
54	Os02g0752200	Q6Z8I7	Os02g0752200 protein	0.37 ± 0.09	ns	ns
65	Os08g0509200	Q84YK7	Beta-glucosidase 27	0.45 ± 0.02	ns	0.57 ± 0.02
121	Os06g0172800	Q5SNC5	Putative seed imbibition protein	2.52 ± 0.32	ns	ns
136	Os03g0340500	Q10LP5	Sucrose synthase 4	5.38 ± 1.07	2.02 ± 0.19	ns
Starch biosynthesis						
6	Os04g0409200	Q0JDF0	Os04g0409200 protein	0.48 ± 0.04	ns	ns
40	Os08g0345800	P15280	Glucose-1-phosphate adenylyltransferase small subunit, chloroplastic/amyloplastic	4.66 ± 0.51	ns	ns
41	Os05g0580000	Q688T8	Glucose-1-phosphate adenylyltransferase	0.21 ± 0.08	0.21 ± 0.08	ns
42	Os01g0130400	Q9LGC6	Putative alpha-glucosidase	0.48 ± 0.04	ns	ns
44	Os01g0633100	Q7G065	ADP-glucose pyrophosphorylase/AGPase	3.36 ± 0.44	ns	ns
45	Os01g0894300	Q0JGZ6	Fructokinase-1	0.53 ± 0.04	ns	ns
46	Os01g0841600	Q8LR75	Triosephosphate isomerase	ns	0.33 ± 0.05	ns
49	Os06g0675700	Q0DA62	Probable alpha-glucosidase Os06g0675700	4.71 ± 1.86	2.51 ± 0.76	ns
84	Os09g0553200	Q93X08	Os09g0553200 protein	3.23 ± 0.63	ns	ns
86	Os05g0482700	Q5KQH5	Putative 2,3-bisphosphoglycerate-independent phosphoglycerate mutase	0.60 ± 0.05	ns	ns
94	Os08g0520900	Q0J4C6	Os08g0520900 protein	3.19 ± 0.38	2.50 ± 0.36	ns
118	Os08g0191433	Q6Z1D6	Putative starch synthase DULL1	2.45 ± 0.16	ns	2.43 ± 0.19
132	Os04g0164900	Q7X834	OSJNBa0019G23.2 protein/pullulanase	20.08 ± 1.58	5.66 ± 0.45	3.84 ± 0.56
152	Os04g0526600	Q0JBL0	Alpha-amylase/subtilisin inhibitor	6.59 ± 0.45	3.02 ± 0.06	ns
158	Os02g0528200	Q6H6P8	Branching enzyme-3/SBE3	2.36 ± 0.36	ns	ns
159	Os06g0726400	Q0D9D0	Os06g0726400 protein/SBE1	15.54 ± 0.72	3.34 ± 0.24	5.00 ± 0.42
Respration (Glycolysis,TCA and Fermentation)						
4	Os01g0905800	Q5N725	Fructose-bisphosphate aldolase	3.99 ± 0.62	ns	ns
27	Os01g0926300	Q5JK10	Os01g0926300 protein	0.37 ± 0.07	ns	ns
30	Os02g0601300	Q6K5G8	Glyceraldehyde-3-phosphate dehydrogenase 3, cytosolic	3.76 ± 0.30	ns	ns
59	Os02g0169300	Q6H6C7	Phosphoglycerate kinase	4.84 ± 0.51	ns	ns
69	Os06g0668200	Q655T1	Phosphoglycerate kinase	2.56 ± 0.42	ns	ns
76	Os10g0478200	Q7XDC8	Malate dehydrogenase, cytoplasmic	3.51 ± 0.12	ns	ns
82	Os08g0191700	Q0J7H9	Lactoylglutathione lyase	3.30 ± 0.40	3.14 ± 1.07	ns
89	Os11g0210500	Q0ITW7	Alcohol dehydrogenase 2	5.38 ± 1.61	ns	ns
104	Os06g0486800	Q0DC43	Formate dehydrogenase	3.25 ± 0.54	2.41 ± 0.74	ns
120	Os08g0545200	Q6ZBH2	Os08g0545200 protein/Sorbitol dehydrogenase	12.37 ± 2.25	2.49 ± 0.24	5.09 ± 0.97
122	Os03g0293500	Q10MW3	Pyruvate decarboxylase 2	3.96 ± 0.28	2.72 ± 1.11	ns
125	Os06g0326400	Q69T78	Pyrophosphate-fructose 6-phosphate 1-phosphotransferase subunit alpha	2.05 ± 0.21	ns	2.27 ± 0.28
127	Os07g0187200	Q7XI14	Probable D-2-hydroxyglutarate dehydrogenase, mitochondrial	4.60 ± 0.81	ns	ns
173	Os04g0486950	Q7XUG1	Malate synthase	5.85 ± 1.70	4.64 ± 0.51	ns
Photosynthesis						
1	Os01g0711000	Q8S7T5	ATP synthase subunit alpha	ns	4.23 ± 1.46	ns
3	Os10g0356000	P0C512	Ribulose bisphosphate carboxylase large chain	2.74 ± 0.09	2.40 ± 0.75	ns
7	Q8S6G5[a]	Q8S6G5	Photosystem II CP43 reaction center protein	ns	1.92 ± 0.09	ns
23	Q69VC8[b]	Q69VC8	Photosystem II CP47 reaction center protein	ns	3.17 ± 1.27	ns
43	Os03g0563300	Q53RM0	Magnesium-chelatase subunit ChlI, chloroplastic	0.46 ± 0.01	ns	ns
57	Os10g0492000	Q9FWV2	Putative chloroplast inner envelope protein	2.36 ± 0.57	ns	ns
Material transport						
22	Os07g0448800	Q8H5N9	Probable aquaporin PIP2–1	ns	3.21 ± 0.71	ns
64	Os08g0513600	Q6Z8M9	Os08g0513600 protein	6.26 ± 2.17	ns	ns
75	Os02g0202400	Q6Z782	Os02g0202400 protein	3.04 ± 0.84	ns	ns
78	Os11g0644100	Q2R0I6	Leucine Rich Repeat family protein, expressed	0.24 ± 0.02	ns	ns
111	Os03g0271200	Q10NF2	Protein TOC75, chloroplastic	4.63 ± 0.36	1.95 ± 0.32	2.45 ± 0.41
123	Os05g0111200	Q65XV6	Os05g0111200 protein	3.77 ± 0.18	2.85 ± 0.23	ns
126	Os03g0240500	Q10PB3	Translocase of chloroplast	2.27 ± 0.17	ns	ns
Signal transduction						
2	Os03g0710800	Q10E23	14–3-3-like protein GF14-F	0.53 ± 0.01	ns	ns
101	Os06g0110100	Q8H684	OSEYA1	4.56 ± 1.10	1.86 ± 0.18	2.51 ± 0.58
129	Os01g0356800	Q0JMV9	Os01g0356800 protein/ GTP binding protein	35.53 ± 10.52	2.45 ± 0.06	12.89 ± 1.52
138	Os02g0799000	Q69QZ0	Probable protein phosphatase 2C 27	5.05 ± 0.74	ns	ns
Stress and defense						
5	Os07g0186000	Q0D840	Thioredoxin H1	ns	3.65 ± 0.82	ns
8	Os02g0115700	Q0E4K1	Catalase isozyme A	0.28 ± 0.01	ns	0.49 ± 0.07
10	Os05g0116100	Q65XA0	Dehydroascorbate reductase	2.15 ± 0.13	2.97 ± 0.40	ns
11	Os05g0323900	Q43008	Superoxide dismutase [Mn], mitochondrial	1.77 ± 0.05	3.29 ± 0.33	ns
21	Os04g0508300	P55142	Glutaredoxin-C6	2.95 ± 0.11	3.44 ± 0.66	ns
24	Os05g0157200	Q75M01	Os05g0157200 protein	ns	2.97 ± 0.28	ns
35	Os12g0244100	Q2QV45	70 kDa heat shock protein	5.12 ± 1.21	2.18 ± 0.62	ns
58	Os05g0453700	Q7XXS5	Os05g0453700 protein	4.37 ± 0.28	3.23 ± 0.16	ns
67	Os07g0624600	Q7XI41	Probable trehalose-phosphate phosphatase 3	0.35 ± 0.03	0.29 ± 0.08	ns
73	Os12g0514500	Q0IN14	Hsp90 protein, expressed	0.36 ± 0.05	0.32 ± 0.05	ns
81	Os01g0270100	Q0JNR2	Cysteine proteinase inhibitor 12	2.14 ± 0.21	ns	ns
113	Os07g0694700	Q0D3B8	Ascorbate peroxidase	0.51 ± 0.06	ns	ns
134	Os01g0663400	Q0JKM8	Os01g0663400 protein	8.82 ± 0.50	2.32 ± 0.43	3.68 ± 0.78
166	Os07g0638300	P0C5C9	1-Cys peroxiredoxin A	14.91 ± 5.52	3.85 ± 0.39	3.59 ± 1.15
174	Os03g0245800	Q10P60	26.7 kDa heat shock protein, chloroplastic	4.84 ± 0.88	2.16 ± 0.53	ns
Protein synthesis and destination						
12	Os04g0685200	Q7XPU2	OSJNBa0088H09.14 protein	0.38 ± 0.04	ns	ns
16	Os06g0687700	Q653F6	Putative t-complex protein 1 theta chain	0.40 ± 0.01	ns	ns
25	Os12g0277500	Q2QU06	60 kDa chaperonin alpha subunit	0.21 ± 0.03	0.23 ± 0.05	ns
34	Os03g0804800	Q75HJ3	Putative TCP-1/cpn60 chaperonin family protein	0.50 ± 0.06	0.61 ± 0.10	ns
48	Os02g0332200	Q6YUK5	T-complex protein 1 subunit delta	0.27 ± 0.03	0.32 ± 0.05	ns
68	Os07g0578300	Q6ZL89	Os07g0578300 protein	0.05 ± 0.01	0.09 ± 0.03	0.38 ± 0.01
71	Os03g0619400	Q6AV23	Putative TCP-1/cpn60 chaperonin family protein	0.40 ± 0.05	ns	ns
79	Os02g0717400	Q6ZGV8	Clustered mitochondria protein homolog	ns	0.30 ± 0.08	ns
95	Os03g0565500	Q10I39	Elongation factor G, mitochondrial, putative, expressed	0.32 ± 0.08	0.33 ± 0.08	ns
103	Os09g0491772	B9G4B3	Os09g0491772 protein	0.16 ± 0.04	0.15 ± 0.04	ns
110	Os02g0100100	Q67IX6	Protein disulfide isomerase-like 1–4	5.62 ± 0.64	ns	3.37 ± 0.67
117	Os01g0185200	Q5VRX8	Os01g0185200 protein	3.21 ± 0.26	ns	ns
119	Os01g0752700	Q5JMX4	Os01g0752700 protein	0.31 ± 0.04	0.28 ± 0.02	ns
130	Os09g0451500	Q67UF5	Protein disulfide isomerase-like 2–3	2.15 ± 0.47	ns	ns
135	Os02g0506500	Q6K6K7	Ubiquitin-like modifier-activating enzyme 5	3.28 ± 1.07	ns	ns
143	Os09g0252800	Q6K3Y7	Putative ubiquitin-protein ligase 1	ns	0.53 ± 0.04	ns
144	Os02g0115900	Q6Z7B0	Dnak-type molecular chaperone Bip	6.30 ± 1.31	ns	3.84 ± 1.04
147	Os05g0557200	Q6I605	Os05g0557200 protein	2.13 ± 0.15	ns	ns
148	Os07g0215500	Q0D7S0	Allergen RA5B	22.26 ± 5.08	4.59 ± 0.37	ns
153	Os07g0213800	Q8H4M4	Allergenic protein	ns	5.68 ± 0.20	ns
155	Os11g0199200	Q53LQ0	Protein disulfide isomerase-like 1–1	4.62 ± 0.40	ns	ns
161	Os03g0610650	Q75H81	Serpin-ZXA	3.53 ± 0.52	2.30 ± 0.33	ns
170	Os05g0519700	Q6F2Y7	Chaperone protein ClpB1	4.54 ± 0.18	2.35 ± 0.39	1.98 ± 0.35
Storage proteins						
97	Os07g0609000	Q6YTX6	Seed protein	19.17 ± 2.41	6.65 ± 2.22	ns
99	Os01g0762500	Q0JJ36	Glutelin	1.54 ± 0.02	ns	ns
128	Os03g0793700	Q852L2	Cupin family protein, expressed	8.40 ± 1.03	4.63 ± 0.42	ns
141	Os05g0499100	Q0DH05	Alpha-globulin	39.03 ± 4.70	4.02 ± 0.47	ns
145	Os02g0242600	Q6ESW6	Glutelin	19.54 ± 5.20	ns	9.51 ± 1.33
151	Os03g0197300	Q0DUA3	Os03g0197300 protein	25.94 ± 10.52	6.59 ± 1.98	ns
157	Os02g0456100	Q6K7K6	Glutelin	36.64 ± 4.06	3.66 ± 0.31	11.87 ± 0.71
163	Os03g0336100	Q0DS36	Os03g0336100 protein	23.65 ± 1.52	3.60 ± 0.61	ns
165	Os02g0249900	Q0E2D2	Glutelin	ns	4.24 ± 0.89	ns
167	Os07g0214300	Q0D7S4	Seed allergenic protein RAG2	12.44 ± 2.51	6.26 ± 0.42	ns
169	Os02g0249000	Q6K508	Glutelin	28.90 ± 5.78	1.86 ± 0.30	14.62 ± 1.20
171	Os03g0663800	Q75GX9	Cupin family protein, expressed	31.11 ± 3.49	ns	ns
172	Os02g0268300	Q0E261	Glutelin	56.76 ± 1.25	4.28 ± 0.71	ns
Amino acid metabolism						
9	Os08g0447000	Q6ZAA5	D-3-phosphoglycerate dehydrogenase	0.18 ± 0.06	0.28 ± 0.11	ns
20	Os11g0216900	Q0ITU1	Methylthioribose-1-phosphate isomerase	0.31 ± 0.01	ns	ns
52	Os03g0738400	Q7Y1F0	Serine hydroxymethyltransferase	1.99 ± 0.05	ns	ns
66	Os03g0223400	Q10PS4	Glutamine synthetase	5.20 ± 0.10	3.93 ± 1.24	ns
70	Os09g0255400	Q8H3R5	Putative indole-3-glycerol phosphate synthase	3.82 ± 0.69	ns	ns
85	Os12g0235800	Q2QVC1	Argininosuccinate synthase, chloroplast, putative, expressed	2.74 ± 0.13	ns	ns
92	Os03g0136200	Q10S41	Methyltransferase	0.42 ± 0.04	ns	ns
93	Os12g0607000	Q2QME6	Homocysteine S-methyltransferase 3	3.83 ± 0.20	ns	ns
105	Os12g0138900	Q2QXY9	2-isopropylmalate synthase B, putative, expressed	2.75 ± 0.17	1.81 ± 0.27	ns
114	Os02g0783625	Q6K7D6	Putative lysine-ketoglutarate reductase/saccharopine dehydrogenase bifunctional enzyme	3.13 ± 0.56	ns	ns
140	Os12g0145100	Q2QXS4	Os12g0145100 protein	ns	ns	2.63 ± 0.68
142	Os10g0390500	Q94HC5	Putative alanine amino transferase	3.07 ± 0.38	ns	3.02 ± 0.44
149	Os12g0578200	Q2QN58	Chorismate mutase, chloroplast, putative, expressed	18.67 ± 2.19	1.92 ± 0.11	8.26 ± 0.90
150	Os03g0171900	Q10R45	Alanine-glyoxylate aminotransferase 2, mitochondrial, putative, expressed	3.68 ± 0.78	ns	ns
160	Os04g0389800	Q0E0Z3	Acetolactate synthase	3.41 ± 0.30	1.80 ± 0.24	ns
162	Os01g0760600	Q0JJ47	Aspartate aminotransferase	3.82 ± 0.36	ns	3.30 ± 0.31
Nucleotides						
29	Os10g0539500	Q7XUC9	Histone H4	0.39 ± 0.03	ns	ns
37	Os01g0550000	Q5JK84	DEAD-box ATP-dependent RNA helicase 15	0.54 ± 0.06	ns	ns
60	Os01g0275600	Q9SDG8	Protein argonaute 4A	0.32 ± 0.06	0.43 ± 0.09	ns
63	Os02g0736400	Q6Z744	Dihydropyrimidine dehydrogenase	2.25 ± 0.17	ns	ns
87	Os03g0158500	Q8H8C1	Putative RNA-binding protein	ns	0.31 ± 0.05	ns
88	Os02g0214500	Q6H8A9	NAC6	4.80 ± 0.64	ns	ns
102	Os07g0471300	Q69UP6	Protein argonaute 18	0.37 ± 0.08	0.41 ± 0.09	ns
107	Os02g0137400	Q6YXY3	Putative splicing factor 3b, subunit 3, 130 kDa	0.56 ± 0.03	ns	ns
109	Os02g0821800	Q6AT27	Putative fibrillarin	0.42 ± 0.08	ns	ns
115	Os07g0212300	Q8H4U7	Mut T-like protein	10.59 ± 1.42	ns	ns
131	Os02g0523500	Q6H547	Os02g0523500 protein	2.18 ± 0.24	ns	1.95 ± 0.34
Lipid metabolism						
28	Os05g0295300	B9FK36	Acetyl-CoA carboxylase 2	0.64 ± 0.01	ns	ns
51	Os11g0558300	Q2R2L5	AMP-binding enzyme family protein, expressed	0.34 ± 0.14	ns	ns
56	Os03g0181500	Q8H7L2	3-ketoacyl-CoA synthase	0.17 ± 0.02	0.25 ± 0.01	ns
62	Os06g0156700	Q5VMA4	Os06g0156700 protein	3.49 ± 0.43	ns	ns
77	Os05g0567100	Q0DFW1	Aspartic proteinase oryzasin 1	2.58 ± 0.49	2.54 ± 0.40	ns
96	Os01g0880800	Q8LJJ9	Stearoyl-[acyl-carrier-protein] 9-desaturase 1, chloroplastic	ns	0.35 ± 0.05	ns
108	Os07g0188800	Q6Z4E4	Methylmalonate semi-aldehyde dehydrogenase	2.13 ± 0.19	2.04 ± 0.40	ns
124	Os06g0260500	Q5Z7E7	3-ketoacyl-CoA synthase	0.24 ± 0.10	0.26 ± 0.12	ns
146	Os01g0348600	Q94CN1	Os01g0348600 protein	0.38 ± 0.15	ns	ns
Secondary metabolism						
31	Os07g0529600	Q7XXS4	Thiamine biosynthetic enzyme	0.30 ± 0.02	0.30 ± 0.02	ns
47	Os08g0157500	Q6ZD89	Flavone 3′-O-methyltransferase 1	4.73 ± 0.80	2.59 ± 0.69	ns
55	Os08g0498400	Q7F8T6	Tricin synthase 2	3.56 ± 0.56	2.19 ± 0.30	ns
116	Os09g0446800	Q0J1E1	Os09g0446800 protein	2.55 ± 0.03	1.67 ± 0.18	ns
139	Os03g0192700	Q10QK8	Inositol-3-phosphate synthase	2.06 ± 0.18	ns	ns
164	Os08g0189100	Q6YZA9	Germin-like protein 8–2	7.61 ± 0.71	5.34 ± 0.86	ns
Unknown						
13	Os12g0555500	Q2QNS7	Os12g0555500 protein	0.49 ± 0.03	ns	ns
15	Os06g0646500	Q67W57	Os06g0646500 protein	ns	ns	0.57 ± 0.04
17	Os03g0278200	Q10N92	Os03g0278200 protein	0.46 ± 0.05	ns	ns
18	Os11g0687100	Q2QZH3	Os11g0687100 protein	0.08 ± 0.03	0.21 ± 0.11	0.31 ± 0.05
32	Os12g0182200	Q2QWU7	Dihydrolipoamide S-acetyltransferase, putative, expressed	0.53 ± 0.05	ns	ns
39	Os06g0613000	Q69WY2	Uncharacterized protein	0.36 ± 0.04	ns	ns
53	Os01g0916600	Q7F2X8	Os01g0916600 protein/OsGRP2	0.24 ± 0.01	0.28 ± 0.01	ns
61	Os11g0687200	Q2QZH2	Expressed protein	0.30 ± 0.05	ns	0.54 ± 0.03
72	Os07g0409100	Q7XTM4	OSJNBa0033G05.21 protein	0.53 ± 0.05	ns	ns
74	Os07g0638100	Q8GVH2	Os07g0638100 protein	0.53 ± 0.03	ns	ns
80	Os01g0128400	Q9LGA3	Os01g0128400 protein	2.00 ± 0.06	ns	ns
83	Os04g0531900	Q7X8W6	OSJNBa0081C01.20 protein	5.73 ± 1.54	4.34 ± 0.33	ns
90	Os10g0463200	Q8H906	Putative early nodulin gene (Enod) related protein	0.36 ± 0.03	0.46 ± 0.04	ns
98	Os03g0327600	Q10M12	Expressed protein	2.03 ± 0.08	ns	ns
100	Os07g0568700	Q0D5C7	Os07g0568700 protein	0.44 ± 0.03	ns	ns
106	Os04g0482800	Q7XUP3	OSJNBb0011N17.20 protein	3.73 ± 0.15	2.14 ± 0.10	ns
112	Os02g0783700	Q0DX00	Os02g0783700 protein	2.05 ± 0.19	ns	ns
133	Os05g0132100	Q0DL03	Os05g0132100 protein	0.34 ± 0.05	ns	ns
137	Os06g0214300	Q69Y21	Os06g0214300 protein	2.85 ± 0.43	1.68 ± 0.16	ns
156	Os04g0404400	Q7X6I8	OJ000315_02.8 protein	16.73 ± 7.88	5.11 ± 1.18	ns
168	Os10g0463800	Q337M4	Os10g0463800 protein	3.60 ± 0.15	2.59 ± 0.29	ns

Accession: the code of the identified protein in RAP database (http://rapdb.dna.affrc.go.jp/); [a] and [b]: the code of these two identified proteins in RAP database were not found; ns means no significant change of protein abundance between the two compared samples; T0: control treatment with no spikelet thinning; T2: the upper 2/3 of spikelets were removed. SS: Superior spikelets; IS: Inferior spikelets; Values are means ± S.D. of two replications. The screening criteria for differentially expressed proteins was a fold change >1.5 or <0.67 and a *p*-value <0.05

### Functional classification of DEPs between SS and IS under different treatments

**Fig. 4:**
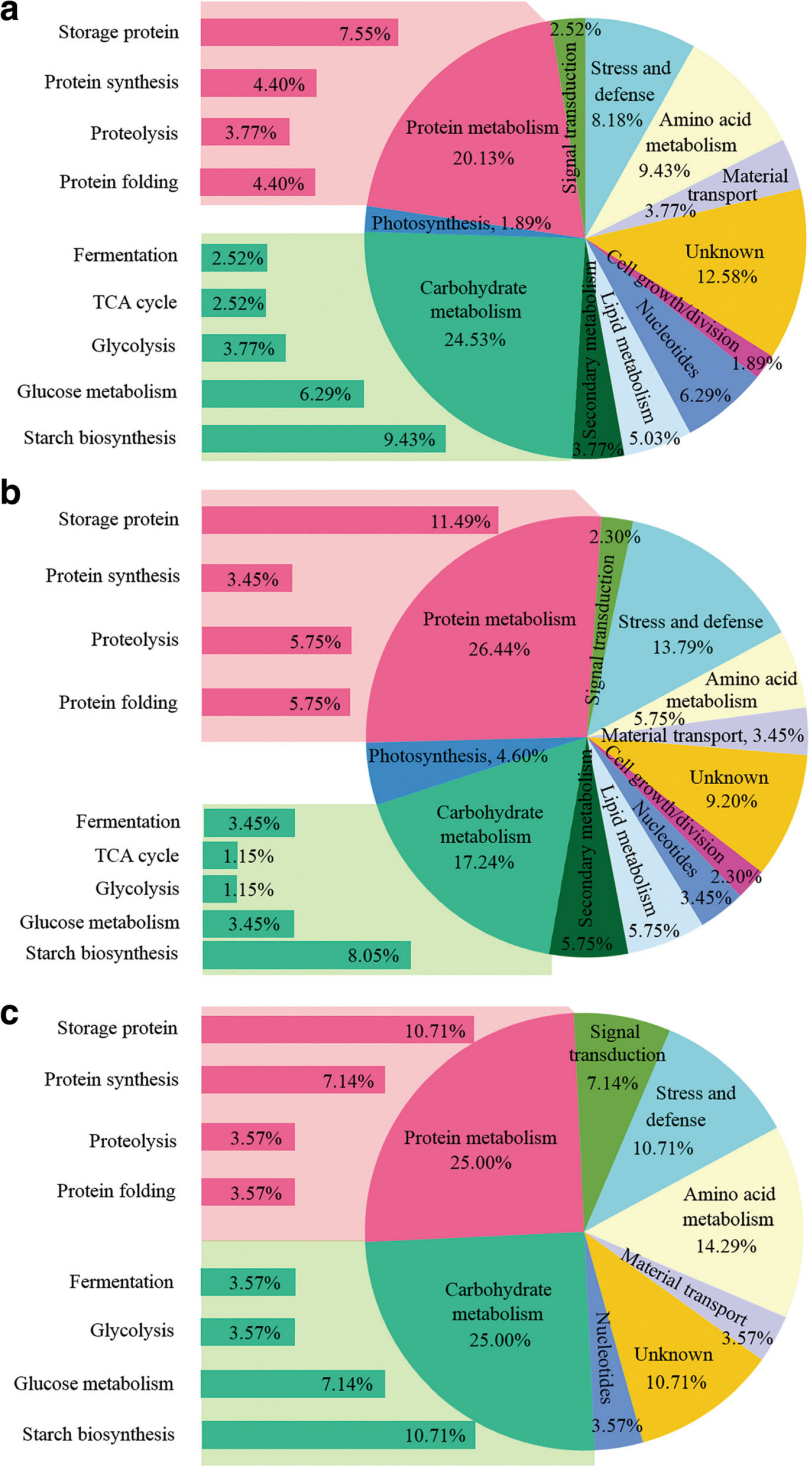
Functional classifications of the differentially expressed proteins in groups **a** (T0-SS / T0-IS), **b** (T0-SS / T2-IS) and **c** (T2-IS / T0-IS). T0 represent control treatment with no spikelet thinning and T2 represent treatment with the upper 2/3 of spikelets were removed

DEPs were classified according to their biological functions and were divided into 11 categories, including carbohydrate metabolism, protein metabolism, secondary metabolism, lipid metabolism, nucleotide metabolism, amino acid metabolism, photosynthesis, cell growth/division, material transport, signal transduction, and stress/defense (Table 2, Fig. 4). In this study, proteins with unknown biological functions or those that could not be attributed to these 11 categories were classified into an unknown protein category. Among the 11 major functional categories, carbohydrate metabolism includes glucose metabolism, starch biosynthesis, glycolysis, tricarboxylic acid (TCA) cycle, and fermentation, while protein metabolism includes protein synthesis, proteolysis, protein folding, and storage.

The T0-SS/T0-IS comparison resulted in the greatest number of DEPs (159), which mainly participated in physiological and biochemical processes including carbohydrate metabolism (24.5%), protein metabolism (20.13%), stress/defense (8.18%), and amino acid metabolism (9.43%) (Fig. 4-a). Relatively fewer DEPs were identified in the T0-SS/T2-IS comparison (87), and these mainly participated in the same metabolic processes as those in the T0-SS/T0-IS comparison. Among these, 17.24% were related to carbohydrate metabolism and 26.44% were associated with protein metabolism (Fig. 4-b). The T2-IS/T0-IS comparison resulted in the fewest DEPs (28), but 25% of these were involved in carbohydrate metabolism and 25% were involved in protein metabolism. Moreover, 7.14% of DEPs in this group were associated with signal transduction, which was significantly more than 2.52% in the T0-SS/T0-IS comparison and 2.3% in the T0-SS/T2-IS comparison (Fig. 4-c).

Together, the above experimental results showed that carbohydrate metabolism and protein metabolism play key roles in the differential development of SS and IS. Among the three comparisons, starch synthesis and protein storage functions were relatively prevalent among carbohydrate metabolism and protein metabolism functions (Fig. 4), suggesting that the supply of carbohydrates to IS increased after SS removing [16], starch and protein synthesis in the grains are significantly enhanced. It is worth noting that the signal transduction function showed the greatest influence on IS development after SS removal, which may be one of the reasons for the increase in IS grain filling after SS removal treatment.

## Discussion

### Physiological differences between SS and IS under different treatments

The phenomena of low seed setting rates and poor plumpness are common in large-panicle rice varieties, and this is mainly due to poor IS grain filling and the formation of empty and blighted grains of rice [31]. These phenomena were also observed in this study. The grain weight and seed setting rate were significant different between SS and IS. SS elongated rapidly and grew well at 10 DPA, while IS were in a state of developmental stagnation. After the removal of SS, the IS grain size and grain weight significantly increased, indicating that 10 DPA was the end of the stagnant grain filling period and the beginning of the grain filling initiation period. Limited assimilate supply was generally considered to be the main cause of poor IS grain filling [7, 11]. The results from our previous studies [16] and this study support this view as well, as SS removal significantly improved IS grain size, sucrose content, grain weight, and grain filling rate in W1844. Since grain filling is a highly complex process, its molecular mechanisms need to be further elucidated.

### Low expression proteins associated with endosperm cell growth and division leading to small sink capacity

A positive correlation between endosperm cell numbers and grain weight has been found in rice [32], wheat [33], and maize [34]. Previous reports showed that SS had a large number of endosperm cells, and thus a large sink size [5]. However, IS endosperm cell division was stagnant at the early grain filling stage, which limited IS sink establishment. In the present study, three cell division-related proteins, actin, annexin, and IAA-amino acid hydrolase ILR1-like 8 were identified. Significant differences in the expressions of these three proteins between SS and IS were considered to be very important for endosperm cell division.

The actin cytoskeleton provides a structural framework for defining cell shape and polarity. Its dynamic properties provide the driving force for cells to move and to divide [35]. Annexins are thought to be associated with cell proliferation and differentiation [36]. In this study, actin and annexin were identifed and their abundances in T0-SS showed significantly higher up-regulation compared with those in T0-IS, this matched well with differences in endosperm cell division between SS and IS. Although SS removal treatment improved IS grain filling, IS grain weight was still lower under T2 than that of T0-SS. Actin, which is involved in endosperm cell division, was 1.76-fold higher in T0-SS than in T2-IS, indicating that compared with T0-SS, sink capacity was smaller in T2-IS, thus explaining the low grain weight in T2-IS at the protein expression level.

Amide-linked conjugates of indole-3-acetic acid (IAA) may serve as reservoirs of inactive IAA that can be hydrolyzed by IAA-conjugate hydrolases, releasing free IAA from the conjugate form. Thus, IAA-conjugate hydrolases are likely to play an important role in regulating free IAA levels [37, 38]. For example, in maize germination, conjugate hydrolysis provides free IAA to the developing seedling [39]. IAA-amino acid hydrolase ILR1-like 8 is an IAA-conjugate hydrolase, and increasing its abundance could elevate levels of free IAA. IAA is an important signal in cereal endosperm development [40]. Low IAA leads to low endosperm cell division in rice IS [41]. Based on these findings, the expression of IAA-amino acid hydrolase ILR1-like 8 may be important for endosperm cell division. Our comparative proteomic results showed that the abundance of IAA-amino acid hydrolase ILR1-like 8 in T0-IS was 3.52-fold lower than that in T0-SS, suggesting that levels of free IAA in T0-IS were lower than those in T0-SS. Thus, T0-IS resulted in poor endosperm cell division, as well as low sink capacity and grain weight. While in T2-IS, the abundance of IAA-amino acid hydrolase ILR1-like 8 was still lower than in T0-SS, and the kernel development of T2-IS also poorer than that in T0-SS. These results indicate that the abundances of actin, annexins, and IAA-amino acid hydrolase ILR1-like 8 in rice are important for the establishment of grain sink.

### Low activities of key enzymes associated with sucrose-starch metabolism leading to poor starch synthesis

Grain filling is actually a process of starch biosynthesis and accumulation [42]. Grain filling materials are transported from the source to the grain mainly in the form of sucrose and are converted to starch through a series of enzymatically catalyzed reactions. Among these, sucrose synthase (SuSase) catalyzes and degrades sucrose to produce uridine diphosphoglucose (UDPG) and fructose, and its activity is an index of the rice sink strength [43]. In this study, the abundances of SuSase in SS were higher than those in IS at 10 DPA, which may be attributed to the high sucrose content of SS that needs to be decomposed. In the T2-IS/T0-IS and T0-SS/T2-IS comparisons, the abundance of SuSase was up-regulated and down-regulated, respectively, consistent with sucrose content. Studies have also shown that sucrose exerts a regulatory effect on SuSase activity [7, 44]. Under SS removal, a large amount of assimilate is supplied to the IS, increasing its sucrose content and inducing an increase in SuSase abundance. Therefore, the improvement in IS grain filling after the removal of SS may be attributed to increased assimilates and a stronger capacity for sugar decomposition.

Many enzymes involved in starch synthesis were identified in this study, such as ADP-glucose pyrophosphorylase (AGPase), starch branching enzyme (SBE), OSJNBa0019G23.2 protein (pullulanase), and putative starch synthase DULL1 (SSS). Among these, AGPase is a key enzyme controlling starch accumulation rate, and its up-regulation can achieve high yields [45]. SBE is a key enzyme controlling amylopectin synthesis, and its enzymatic activity is significantly positively correlated with the amylopectin accumulation rate [46], while SSS plays an important role in amylose synthesis [47]. In this study, compared to T0-IS at 10 DPA, the protein abundances of AGPase, SSS, SBE, and pullulanase in T0-SS were up-regulated. This result is consistent with the proteomic results from Zhang et al. [48]. Futhermore, compared to levels in T0-IS, alpha-glucosidase (AGS), which is involved in starch hydrolysis, was down-regulated in T0-SS, while the alpha-amylase/subtilisin inhibitor (ASI), involved in the inhibition of starch hydrolysis, was up-regulated in T0-SS. This facilitated starch accumulation in SS. In general, the results from this study and from previous studies [49] show that reduced activity of the enzymes associated with starch synthesis is the main reason for poor IS grain filling.

**Fig. 5:**
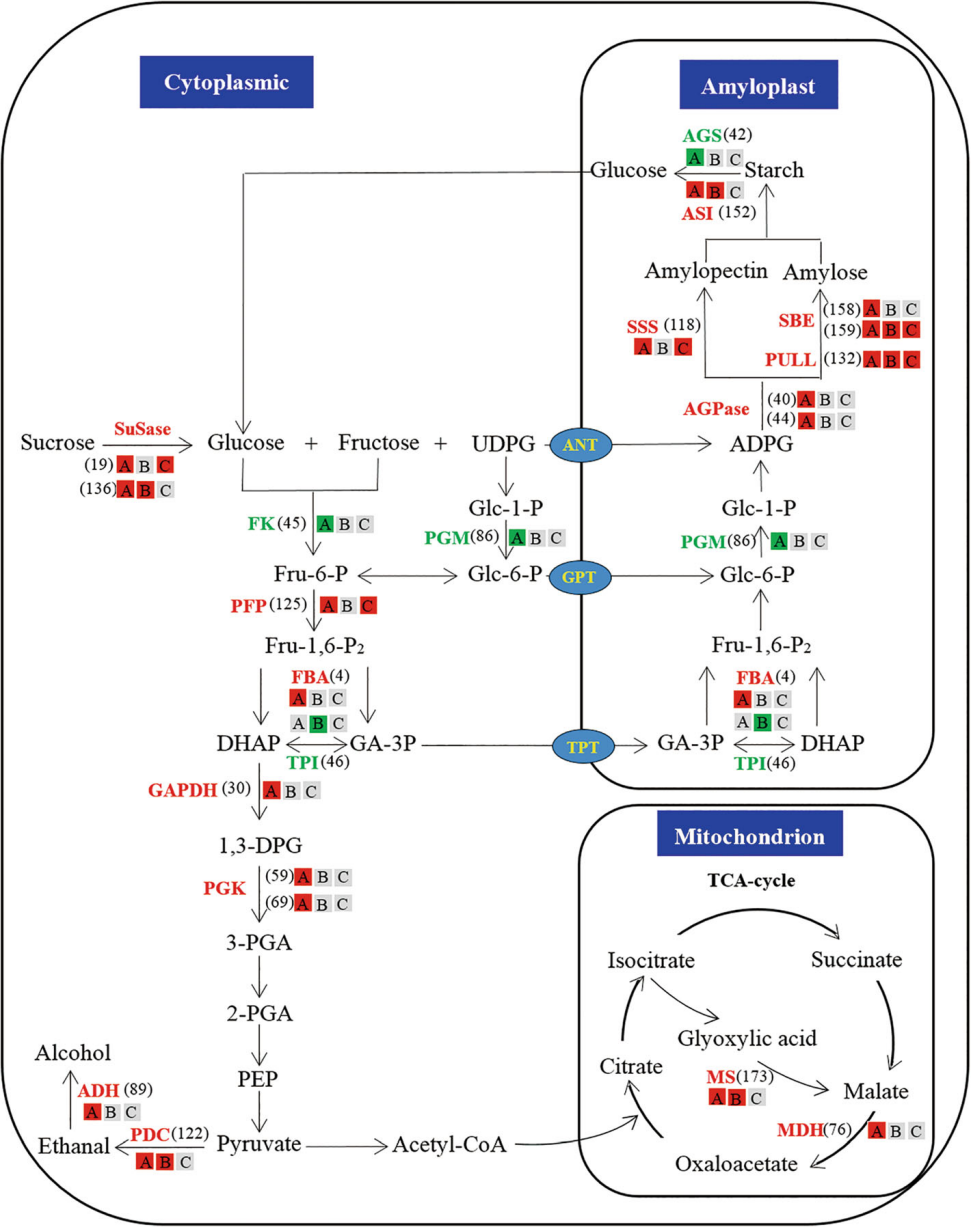
The differentially expressed proteins onto carbohydrate metabolism of rice grain. **Note**: A, B and C represent the groups of T0-SS / T0-IS, T0-SS / T2-IS and T2-IS / T0-IS. *Red* and *green* indicate *p* ≤ 0.05 (*red* denotes significant up-regulation in the endosperm, *green* significant down-regulation); *Light grey* indicate no significant difference in the level of *p* ≤ 0.05. SuSase, sucrose synthase; SBE, starch branching enzyme; UDPG, uridine diphosphate glucose; ADPG, adenosine diphosphate glucose; DHAP, dihydroxyacetone phosphate; GA-3P, glyceraldehyde-3-phosphate; 1,3-DPG, 1,3-diphosphoglycerate; 3-PGA, 3-phosphoglycerate; 2-PGA, 2-phosphoglycerate; PEP, phosphoenolpyruvate; FK, fructokinase; PFP, pyrophosphate-fructose 6-phosphate 1-phosphotransferase; PGM, phosphoglucomutase; AGPase, adenosine diphosphoglucose pyrophosphprylase; PULL, pullulanase; DULL1, putative starch synthase DULL1; FBA, fructose-bisphosphate aldolase; TPI, triosephosphate isomerase; AGS, alpha-glucosidase; ASI, alpha-amylase/subtilisin inhibitor; GAPDH, glyceraldehyde-3-phosphate dehydrogenase; PGK, phosphoglycerate kinase; PDC, pyruvate decarboxylase; ADH, alcohol dehydrogenase; MS, malate synthase; MDH, malate dehydrogenase, cytoplasmic; ANT, adenylate transporter; GPT, glucose phosphate translocator; TPT, triose phosphate translocator

As Fig. 5 shows, the DEPs related to carbohydrate metabolism in the T2-IS/T0-IS comparison mainly participate in starch synthesis. Compared to T0-IS, the abundances of SSS, SBE, and pullulanase in T2-IS were all up-regulated, which may be due to the increasing supply of sucrose to the IS after SS removal. Similar to the findings of previous studies, we showed that expressions of SSS and SBE were up-regulated by an increase in sucrose [50]. Therefore, improving IS grain filling after SS removal may be achieved through an increase in the sucrose content, which in turn induces the up-regulation of SBE and SSS, thus promoting starch synthesis in IS.

### Weakened photosynthesis and respiration resulting in stagnation of grain development

Carbohydrate metabolism mainly includes glycolysis and the TCA cycle, which provides energy and material for the transformation and synthesis of metabolites [51]. In this study, proteins associated with carbohydrate metabolism were identified (Fig. 5), including proteins involved in glycolysis, such as fructose-bisphosphate aldolase (FBA), glyceraldehyde-3-phosphate dehydrogenase 3 (GAPDH), lactoylglutathione lyase, and phosphoglycerate kinase (PGK), as well as proteins participating in the TCA cycle, such as malate dehydrogenase (MDH). The abundances of these proteins were lower in IS than in SS, which is consistent with the results of Zhang et al. [48]. Reduced glycolysis and TCA cycle activity in the IS at 10 DPA is not able to supply enough material and energy for cell expansion and starch synthesis, and thus affects the formation of the grain sink. Under hypoxic conditions, enzymes in the alcohol fermentation pathway are important for the formation of ATP, which is required to maintain starch synthesis, including pyruvate decarboxylase 2 (PDC 2) and alcohol dehydrogenase 2 (ADH2). The abundances of these two enzymes in the IS were lower than those in the SS, and this indicated that the down-regulation of alcohol fermentation in the IS resulted in a decrease in ATP, thus affecting the initiation of IS grain filling and restricting normal starch synthesis.

We also identified other proteins associated with energy metabolism, such as D-2-hydroxyglutarate dehydrogenase (D-2HGDH), which catalyzes the formation of 2-ketoglutarate from D-2-hydroxyglutarate in the mitochondria and releases energy [52], and formate dehydrogenase (FDH), which catalyzes the oxidation of formic acid to CO_2_ and reduces NAD^+^ to NADH [53, 54]. Our proteomic study indicated that these two proteins were down-regulated in the IS at 10 DPA. Ribulose bisphosphate carboxylase large chain (Rubisco), a key enzyme for CO_2_ fixation during plant photosynthesis [55], was identified in the present study, and its abundance in SS was 2.74-fold higher than that in IS. It may be inferred therefore that photosynthesis was more productive in SS than in IS, producing more carbohydrates used for grain filling. The reduced abundances of these energy metabolism-related proteins in the IS therefore explains poor IS grain filling at the proteomic level.

Pyrophosphate-fructose 6-phosphate 1-phosphotransferase (PFP) can reversibly catalyze the conversion between fructose-6-phosphate (F6P) and fructose 1,6-bisphosphate (F-1,6-P_2_) by phosphorylation and dephosphorylation [56]. However, in vivo, the positive reaction from F6P to F-1,6-P_2_ is catalyzed by the irreversible enzyme phosphofructokinase (PFK). It is worth noting that, unlike PFK, the positive reaction catalyzed by PFP does not require consumption of ATP. Therefore, in higher plants, oxygen-free glycolysis is dependent on PFP, which is more economical from the standpoint of energy transformation. PFP also stores energy in a PPi (phosphate group) from the perspective of gluconeogenesis [57]. In this study, we found that the PFP abundance of T2-IS was 2.27-fold higher than that of T0-IS, which demonstrated that PFP plays an important role in the processes of glycolysis and gluconeogenesis in IS after SS removal and that its activity is conducive to the economical utilization of energy.

### Low abundances of proteins associated with proteins metabolism (protein synthesis, folding, and storage) leading to poor protein synthesis

Rice protein formation is closely related to the nitrogen nutritional status of the plant [58], which is regulated by nitrogen metabolism. Transamination is a crucial process of nitrogen metabolism, that involves a variety of enzymes, including aspartate amino transferase (GOT) and alanine amino transferase (GPT). In higher plants, inorganic nitrogen is converted to amino acids by catalysis with these two transaminases, thus providing a variety of amino acid donors for the synthesis and metabolism of grain proteins [59]. In this study, the abundances of GOT and GPT in SS were 3.82-fold and 3.07-fold higher, respectively, than those in IS, which may be due to the fact that in the early filling stage, less material was supplied to the IS, resulting in poor nitrogen metabolism. A proteomic study by Zhang et al. [48] has shown that the abundances of GPT in IS are down-regulated, probably owing to the lack of nitrogen and accelerated aging of the rice plants at later stages. The results also showed that the abundances of GOT and GPT in T2-IS were higher than those in T0-IS, which suggested that IS grain filling was improved after SS removal, probably due to the up-regulation of GPT and GOT, promoting IS protein formation.

Molecular chaperones are effective in regulating the proper folding of polypeptide chains, thereby forming active proteins [60]. In this study, molecular chaperones, such as the DnaK-type molecular chaperone Bip and the chaperone protein ClpB1, were found to be differentially expressed between SS and IS. The abundances of these two proteins in T0-SS were 6.30-fold and 4.54-fold higher than those in T0-IS, while they were all higher in T2-IS than those in T0-IS. Thus, well-developed rice grains probably require high abundances of molecular chaperones, which regulate the proper folding of polypeptide chains.

The formation or isomerism of disulfide bonds plays an important role in protein folding and metabolic regulation [61]. Protein disulfide isomerase (PDI) and protein disulfide isomerase-like (PDILs) can catalyze the formation of disulfide bonds in proteins [62]. Shimoni et al. [63] was the first to report that PDI was involved in the folding of storage proteins during endosperm formation. In this study, the abundances of PDIL1–1, PDIL1–4, and PDIL2–3 in SS were all higher than those in IS. Johnson et al. [64] demonstrated that in wheat, PDIL1–1 was essential for accurate assembly and distribution of gliadin and glutelin in the endoplasmic reticulum. Moreover, PDIL1–1 was found to control endosperm development by regulating the quantity and composition of proteins in rice seeds [65]. The results of this study indicated that the synthesis of storage proteins in SS is elevated compared to that in IS during the formation of the seed endosperm, and this may be one of the reasons for poor IS grain filling. Additionally, the abundance of PDIL1–4 in T2-IS was higher than that in T0-IS, which may be attributed to a significant increase in the nitrogen compounds supplied to IS after SS removal. The quantity of storage proteins in IS was increased by up-regulating PDIL abundance during the formation of the seed endosperm, thereby improving IS grain filling.

Storage proteins are mainly found in the rice endosperm and can be divided into glutelin, globulin, albumin, and prolamin according to their solubility. The contents and proportions of these proteins affect the quality of rice. In this study, we identified a large number of differentially expressed storage proteins between SS and IS, such as glutelin, globulin, and vegetative storage proteins (cupin family protein). The abundances of glutelin (Nos. 145, 157, 169) were 9.51-fold, 11.87-fold, and 14.62-fold higher in T2-IS than those in T0-IS, respectively. Ma et al. [66] showed that high-temperature stress significantly increased glutelin abundance in rice grains, but there was no effect of the application of panicle fertilizer. Dong et al. [22] suggested that drought stress may change the abundances of storage proteins in rice grains. The results of this study showed that during grain filling, SS removal could also affect the abundance of glutelin in the IS, though the specific regulatory mechanism involved requires further study.

### GTP binding protein, PP2C and IAA-amino acid hydrolase ILR1-like 8, in signaling networks involved in IS development

The growth and development of plants are mainly regulated by genetic and environmental information. The transmission of changing environmental information, namely, cellular signal transduction, regulates carbohydrate and energy metabolisms, as well as physiological and biochemical reactions. GTP binding protein participates in a series of signal transduction process in cells, such as the signal transduction of transmembrane messengers, light signal transduction, protein biosynthesis, and cytoskeletal structure formation [67]. In this study, the abundance of GTP-binding protein was 35.53-fold higher in T0-SS than that in T0-IS, indicating that the rate of signal transduction in T0-SS was higher than that in T0-IS. In addition, the abundance of GTP-binding protein in T2-IS was also increased, and it was 12.89-fold higher compared to that in T0-IS, which may be one of the reasons for the improvement in IS grain filling after SS removal.

Protein phosphorylation/dephosphorylation is one of the most important methods of biological signal transmission, and it occurs mainly through the activities of two types of protein with mutually antagonistic biochemical properties: protein kinases and protein phosphatases. Protein phosphatase 2C (PP2C) plays an important role in biological signal transduction and is involved in various ABA signaling pathways in higher plants [68]. ABA is a key hormone involved in the regulation of grain filling, and ABA levels are significantly positively correlated with the grain filling rate [4]. In this study, the abundance of PP2C was 5.05-fold higher in SS than that in IS. Therefore, we suspect that poor IS grain filling may be associated with poor ABA signal transduction. The increase in IAA-amino acid hydrolase ILR1-like 8 abundance may increase the active IAA level in grains. Seth et al. [69] argued that IAA as a signaling substance could control grain growth by regulating the distribution of assimilation products. The main role of IAA in grain filling is to increase the “pull” of its position to assimilates, so that assimilates are supplied primarily to locations with high IAA levels [70]. In this study, the abundance of IAA-amino acid hydrolase ILR1-like 8 in T0-SS was higher than that in T0-IS, suggesting that assimilates were preferentially supplied to the SS and that IS are unable to obtain a timely supply of nutrients after fertilization, resulting in a relative lag in IS grain filling.

### Increased abundance of 14–3-3 protein in IS inhibits starch synthesis

In the process of plant development, 14–3-3 proteins participate in plant signal transduction, substance metabolism, stress response, and other regulatory processes by interacting with other proteins [71]. In recent years, great progress has been made in the study of plant 14–3-3, and it was found that 14–3-3 also plays an important role in starch metabolism. A high content of 14–3-3 proteins in the wheat endosperm inhibits the activity of sucrose synthase [72]. In *Arabidopsis*, inhibition of 14–3-3 activity leads to an increase in starch accumulation [73]. These results suggest that 14–3-3 may inhibit starch synthesis. In this study, the abundance of 14–3-3 protein (14–3-3-like protein GF14-F) in IS was higher than that in SS, consistent with the results from previous studies [48]. Therefore, the higher abundance of 14–3-3 protein in IS could be an important factor leading to the poor development of IS.

### Unknown proteins

Through bioinformatics comparison, we identified many unknown proteins, such as Nos. 83, 106, 156, and 168, which were over 3-fold up-regulated in T0-SS/T0-IS and T0-SS/T2-IS at 10 DPA. However, even using bioinformatics analysis methods, the roles of these proteins could not be identified, and their functions remain unclear.

## Conclusions

A large-panicle japonica rice line W1844 was suitable to explore the physiological and molecular mechanism of poor IS grain filling, for there exists great difference in kernel development between SS and IS. Compared with SS, the IS exhibited more weaker endosperm cell division and lower activity of key enzymes related to sucrose-starch metabolism, carbohydrate metabolism and nitrogen metabolism. In addition, the weakened photosynthesis and respiration could not timely provide enough materials and energy for cell expansion and grain filling, which may result in the stagnation of IS development. Moreover, a higher abundance of 14–3-3 protein in IS could be involved in the inhibition of starch synthesis. However, the removal of SS significantly improved IS grain filling primarily by increasing carbohydrate supply, which increased the activities of key enzymes involved in sucrose-to-starch metabolism and nitrogen metabolism, promoting the starch and protein synthesis. Additionally, the energy metabolism was also improved as the more carbohydrate in IS. Therefore, we argued that a limitation in the assimilate supply may be the main cause of poor IS grain filling. The poor IS grain filling is a complex process and this is confirmed by the proteomic analysis in present study. An integrated method using a combination of omics platforms such as metabolomic and transcriptomic will be needed to understand this mechanism comprehensively.

## Additional file

Additional file 1: List of all proteins identified and quantified by UPLC/MS/MS. (XLSX 59 kb)

## Abbreviations

2-DE: two-dimensional gel electrophoresis
ABA: Abscisic acid
ADPG: Adenosine diphosphate glucose
AGPase: ADP-glucose pyrophosphorylase
DEPs: Differentially expressed proteins
DPA: Days post anthesis
GOT: Aspartate amino transferase
GPT: Alanine amino transferase
IAA: Indole-3-acetic acid
IS: Inferior spikelets
ITRAQ: Isobaric tags for relative and absolute quantitation
MS: Medium spikelets
PFP: Pyrophosphate-fructose 6-phosphate 1-phosphotransferase
PULL: Pullulanase
SBE: Starch branching enzyme
SS: Superior spikelets
SSS: Soluble starch synthase
SuSase: Sucrose synthase
TCA: Tricarboxylic acid
UDPG: Uridine diphosphoglucose
